# Comparative experimental study of the biomechanical properties of retrograde tibial nailing and distal tibia plate in distal tibia fracture

**DOI:** 10.3389/fbioe.2024.1322043

**Published:** 2024-02-20

**Authors:** Xuping Lin, Cong Zhang, Yanfang Yang, Wencheng Yang, Xiaomeng Wang, Haichuan Lu, Qingjun Liu

**Affiliations:** ^1^ Department of Spinal Surgery, Longyan First Affiliated Hospital of Fujian Medical University, Longyan, China; ^2^ Department of Orthopedic Surgery, The Affiliated Dongnan Hospital of Xiamen University, Zhangzhou, China; ^3^ Shengli Clinical Medical College of Fujian Medical University, Fuzhou, China

**Keywords:** distal tibia fracture, internal fixation, retrograde tibial nailing, distal tibia plate, biomechanics

## Abstract

**Objective:** A biomechanical comparative analysis was conducted to evaluate the retrograde tibial nailing (RTN) and distal tibia plate techniques for the treatment of distal tibia fractures.

**Methods:** Fourteen fresh adult tibia specimens were selected, consisting of seven males and seven females aged 34–55 years. The specimens were randomly divided into two groups (Group A and Group B) using a numerical table method, with seven specimens in each group. Group A underwent internal fixation of distal tibial fractures using RTN, while Group B received internal fixation using a plate. The axial compression properties of the specimens were tested in the neutral positions under pressures of 100, 200, 300, 400, and 500 N. Additionally, the torsional resistance of the two groups was assessed by subjecting the specimens to torques of 1.0, 2.0, 3.0, 4.0, and 5.0 N m.

**Results:** At pressures of 400 and 500 N, the axial compression displacement in Group A (1.11 ± 0.06, 1.24 ± 0.05) mm was significantly smaller than that in Group B (1.21 ± 0.08, 1.37 ± 0.11) mm (*p* = 0.023, 0.019). Moreover, at a pressure of 500 N, the axial compression stiffness in Group A (389.24 ± 17.79) N/mm was significantly higher than that of the control group (362.37 ± 14.44) N/mm (*p* = 0.010). When subjected to torques of 4 and 5 N m, the torsion angle in Group A (2.97° ± 0.23°, 3.41° ± 0.17°) was significantly smaller compared to Group B (3.31° ± 0.28°, 3.76° ± 0.20°) (*p* = 0.035, 0.004). Furthermore, at a torque of 5 N m, the torsional stiffness in Group A (1.48 ± 0.07) N m/° was significantly higher than that in Group B (1.36 ± 0.06) N·m/° (*p* = 0.003).

**Conclusion:** The results obtained from the study demonstrate that the biomechanical performance of RTN outperforms that of the distal tibial plate, providing valuable biomechanical data to support the clinical implementation of RTN.

## Introduction

Distal tibia fractures account for approximately 40% of all long bone fractures in the body, with far distal tibia fractures making up approximately 15% of the total cases of distal tibia fractures. (Huebner et al., 2014; Wennergren et al., 2018).

The etiology of distal tibial fractures in young adults is often linked to high-energy trauma, while in older adults, low-energy trauma is a more prevalent causative factor (Wennergren et al., 2018). Treatment options for distal tibia fractures include internal fixation with plate osteosynthesis, minimally invasive plate osteosynthesis (MIPO) or intramedullary nailing (Pairon et al., 2015). The utilization of plates in distal tibial fractures is associated with inherent complications, primarily attributable to the limited soft tissue coverage in this region (Menck et al., 1992). Plate osteosynthesis in the distal tibia may compromise vascularization and soft tissue integrity, necessitating a meticulous evaluation of its appropriateness in patients already vulnerable in this anatomical area (Vallier et al., 2008). Various preexisting factors, encompassing injury-related aspects and prior surgical interventions, collectively contribute to compromised healing and escalate the susceptibility to complications such as cortical necrosis, delayed or non-union, and infection (Dickson et al., 1994; Singer et al., 1998).

MIPO has emerged as a prominent treatment modality for distal tibia fractures (Zou et al., 2013). It is often commended for its minimally invasive approach, employing low-profile and anatomically pre-contoured implants. However, clinical series have reported a noteworthy incidence of implant prominence and soft tissue irritation, ranging from 32% to 52% (Lau et al., 2008; Cheng et al., 2011). In contrast, as an alternative technique, intramedullary nailing not only demonstrates favorable mechanical properties but also offers biological advantages by preserving fracture site vascularity and soft tissue integrity. Nonetheless, antegrade intramedullary nailing of distal tibia fractures presents significant challenges and is associated with the risk of primary and secondary malalignment. Current literature has demonstrated that the optimal approach to treating distal tibia fractures remains a subject of debate (Vallier et al., 2011; Iqbal and Pidikiti, 2013). Retrograde tibial nailing (RTN) represents a novel surgical alternative in the management of distal tibial fractures (Peng et al., 2022). Notably, the adoption of RTN in routine clinical practice has been limited, with only a handful of documented cases in the current literature (Gehr et al., 2004). Given a suitable implant, a closed surgical approach utilizing retrograde tibial nailing appears as an appealing alternative due to its potential to provide stable fracture fixation with minimal additional soft tissue injury and knee preservation. However, before the advent of the RTN in 2017, there existed a dearth of dedicated surgical implants explicitly crafted for this application (Kuhn et al., 2014a).

The RTN presents notable advantages in terms of biomechanical stability for distal tibia fractures, owing to its distal insertion site and multidirectional triple-distal interlocking mechanism. Furthermore, the inclusion of an end cap enables the creation of an angle-stable distal locking screw to nail construct. The RTN demonstrates efficacy across a broad spectrum of distal tibial fractures (Kuhn et al., 2014b), while also preserving local soft tissues and vascularization (Kuhn et al., 2014a).

Fractures occurring in the distal end of the tibia frequently manifest as open fractures, primarily due to inadequate soft tissue protection on the medial aspect of the tibia. Consequently, the application of medial plating is often contraindicated or considered relatively contraindicated in such cases. In contrast, the lateral side of the tibia features relatively abundant soft tissue, rendering lateral plating a safer alternative with a reduced risk of infection. Within our hospital, lateral plating stands out as a commonly employed method for internal fixation in cases of distal tibial fractures. As a result, we have elected to undertake a comparative analysis of both fixation methods in the context of this study.

The purpose of this study was to conduct an exploratory analysis to evaluate the biomechanical performance of RTN compared to the distal tibia plate. The study hypothesized that conducting comparative biomechanical testing between the RTN and distal tibia plate osteosynthesis would demonstrate superior biomechanical properties of the former, particularly regarding axial compression and torsional in an extra-articular distal tibia fracture model. The aim was to provide more robust evidence to support the clinical use of RTN.

## Materials and methods

### Specimen preparation

A total of 14 fresh adult tibial specimens were included in this study, with all surrounding soft tissues cleanly stripped. These specimens included 7 males and 7 females, with an age range of 34–55 years and a mean age of 42 years. Seven specimens were obtained from the right side and seven from the left side. All fresh limb specimens were obtained from the Xiamen University Anatomy Research Institute, with approval from the institutional ethics committee for the use of human cadaveric specimens. Inclusion criteria were as follows: all specimens exhibited normal and intact external morphology. X-ray examination was performed to confirm normal and intact bone quality, absence of tumors, deformities, and skeletal disorders such as osteoporosis. Exclusion criteria included a history of fractures, osteomyelitis, or severe degenerative changes in the specimens. The 14 tibial specimens were randomly divided into two groups using a digital table. Group A consisted of 4 right-sided and 3 left-sided specimens, which underwent internal fixation of distal tibial fractures using RTN. Group B consisted of 3 right-sided and 4 left-sided specimens, which underwent tibial plateau fixation using distal tibial steel plates.

### Model preparation

For the model preparation of both groups, tibial specimens with the surrounding tissues already carefully removed were selected. At a distance of 4 cm from the tibial joint in the distal region, an artificial tibial fracture was created by using a wire saw to cut through the bone.

**FIGURE 1:**
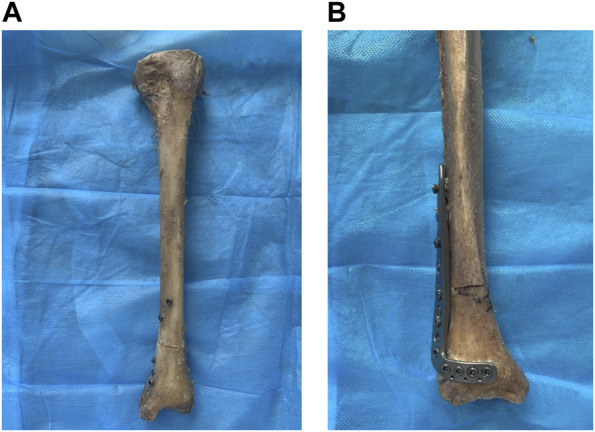
The internal fixation of distal tibia fractures was performed on two groups of specimens. Notes: **(A)** The retrograde tibial nail was used for the internal fixation of distal tibia fractures. **(B)** The distal tibia steel plate was used for the internal fixation of distal tibia fractures.

In Group A, RTN was performed (RTN; Mizuho^®^, Japan). A Kirschner wire was inserted into the medullary cavity of the tibia, approximately 1 cm above the medial malleolus, and an incision was made to expose the medial malleolus under a protective sleeve. An appropriately-sized RTN was then gradually rotated and inserted according to the size of the tibia. With the assistance of a targeting device, locking screws were sequentially placed in the proximal and distal regions (He et al., 2023). Finally, the RTN cap screw was tightened to complete the RTN procedure (Figure 1A).

**FIGURE 2:**
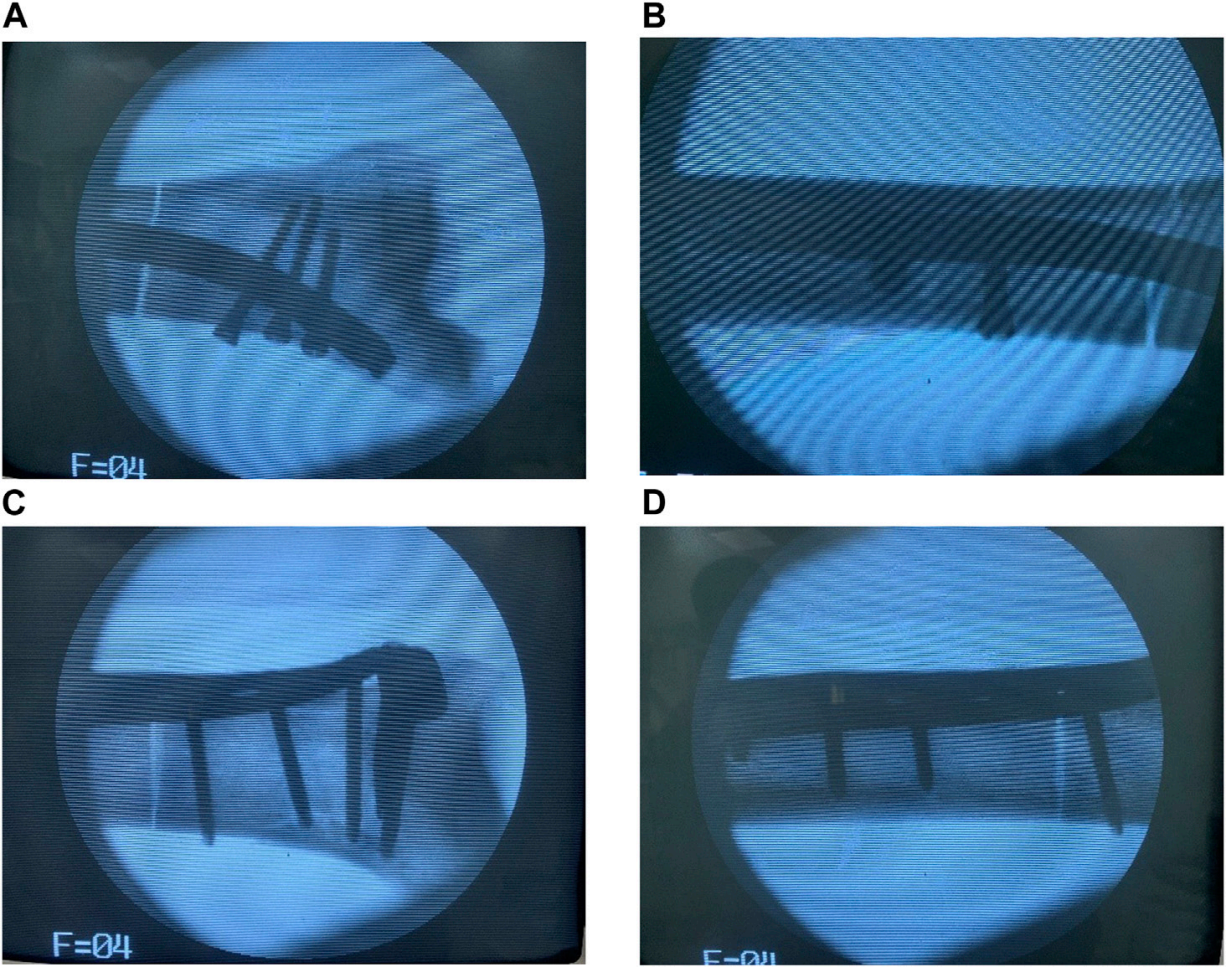
Postoperative X-ray fluoroscopy was performed on the specimens in both groups. Notes: **(A,B)** The retrograde tibial nail was used for the internal fixation of distal tibia fractures. **(C,D)** The distal tibia steel plate was used for the internal fixation of distal tibia fractures.

In Group B, tibial fixation was achieved using a distal tibial L-shaped steel plate. The distal tibial L-shaped steel plate is an external lateral steel plate for the distal end of the tibia produced by Xiamen Double Medical Material Co., Ltd., China. An appropriate plate size was selected based on the size of the tibia and the location of the fracture line. After fracture reduction, the steel plate was placed on the lateral side of the distal tibia, and fixation was achieved using screws of suitable length. The standard surgical technique for plate fixation was followed (Figure 1B). Finally, self-setting dental plaster was used to embed and fix the tibial plateau and distal tibia [8]. This completed the production of the models with tibial distal fractures treated by internal fixation in both groups. Post-operatively, an X-ray fluoroscopy was performed to assess the position and length of the internal fixation devices, as well as the reduction of the fracture (Figure 2).

### Biomechanical testing

We performed biomechanical tests to evaluate the characteristics of the two types of implants. The biomechanical experiments were conducted on the Electroforce 3,510 Bose biomechanical testing machine and the Win Test digital control system (Bose Corp.). Before conducting the biomechanical tests, a preload (at 1/10 of the load) was applied to all specimens to mitigate the potential impact of tibial relaxation and creep on the experimental outcomes.

**FIGURE 3:**
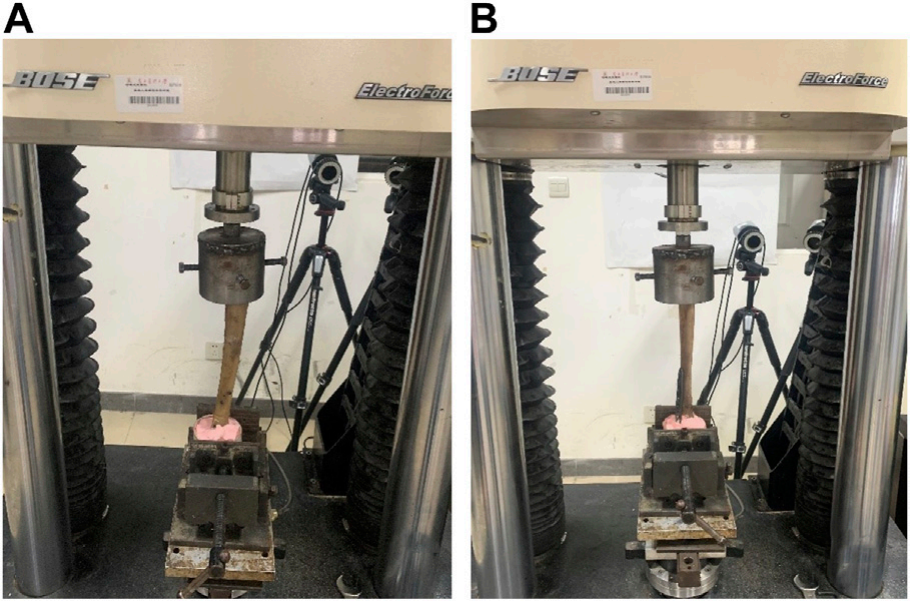
Schematic diagrams of the axial compression and torsion tests. Notes: **(A)** The retrograde tibial nail was used for the internal fixation of distal tibia fractures. **(B)** The distal tibia steel plate was used for the internal fixation of distal tibia fractures.

An axial compression test was conducted. The model was placed in a neutral position on the biodynamic testing machine, with its two ends fixed to the ends of the machine. The initial pressure was set to 0 N, and the axial pressure was increased at a rate of 100 N/s, with the maximum pressure set at 500 N. The axial compression displacement at the tibia fracture site was recorded when the pressure was at 100 N, 200 N, 300 N, 400 N, and 500 N. The axial compression stiffness was calculated at a pressure of 500 N. Each specimen was tested four times, with an interval of 5 min between every two tests, and the average value was taken as the final result (Figure 3). The axial compression stiffness (N/mm) was calculated as follows: load (N)/axial compression displacement (mm).

A torsion test was performed by placing the model in a neutral position on the biodynamic testing machine, with its two ends fixed to the ends of the machine. The pressure was set to 500 N and continuously applied throughout the test. The initial torque was set at 0 N m, and the torque was increased at a rate of 1.25 N m/s, with the maximum torque set at 5.0 N m. During the test, the torsion angle of the specimen was recorded when the torque was at 1.0 N m, 2.0 N m, 3.0 N m, 4.0 N m, and 5.0 N m. The torsion stiffness was calculated when the torque was at 5.0 N m. Each specimen was tested four times, with an interval of 5 min between every two tests, and the average value was taken as the final result (Figure 3). The torsion stiffness (N·m/°) was calculated as follows: torque (N·m)/torsion angle (°).

The data were analyzed using SPSS 26.0 statistical software. Normally distributed continuous variables were presented as mean ± standard deviation (x ± s). The Student’s t*-test* was conducted for the comparison between groups. An error level of less than 5% (*p*-value, 0.05) was defined as statistically significant.

## Results

Both groups successfully prepared models of distal tibial fractures after internal fixation surgery. Intraoperative X-ray fluoroscopy showed satisfactory reduction of the distal tibial fracture, appropriate size and position of the internal fixation device, and proper placement and length of the screws. Subsequent biomechanical testing was carried out smoothly.

### Axial compression test

**FIGURE 4:**
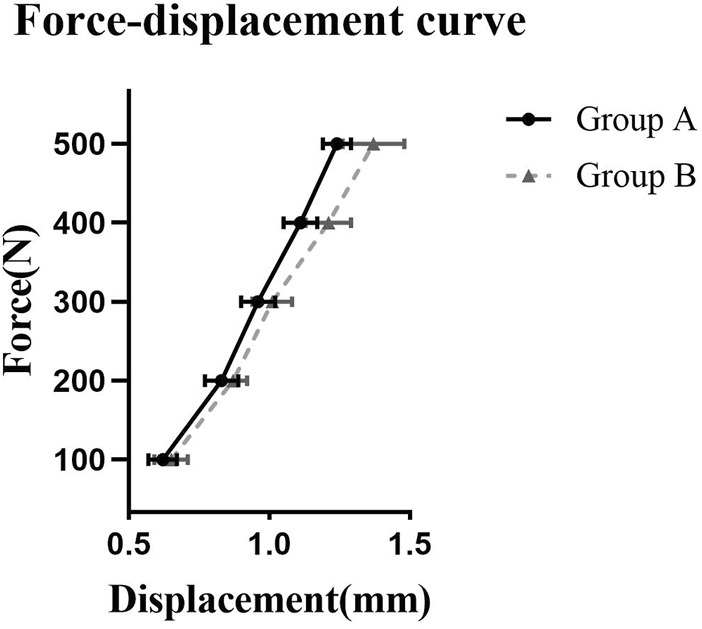
The force-displacement curve for axial compression test. Note: Group A underwent retrograde tibial nailing, Group B underwent distal tibial plating.

At pressure levels of 100 N, 200 N, and 300 N, there was no statistically significant difference in axial compression displacement between the two groups (*p* > 0.05). However, at pressure levels of 400 N and 500 N, Group A exhibited significantly lower axial compression displacement compared to Group B (*p* < 0.05). Furthermore, at a pressure level of 500 N, Group A demonstrated a higher axial compression stiffness than Group B, and the difference was statistically significant (*p* < 0.05). Refer to Table 1; Figure 4 for detailed results.

**TABLE 1:** Axial compression test between the two groups at different pressures.

	Axial compression displacement (mm)					Axial compression stiffness (N/mm)
	100 N	200 N	300 N	400 N	500 N	
Group A	0.62 ± 0.05	0.83 ± 0.06	0.96 ± 0.06	1.11 ± 0.06	1.24 ± 0.05	389.24 ± 17.79
Group B	0.65 ± 0.06	0.87 ± 0.05	1.01 ± 0.07	1.21 ± 0.08	1.37 ± 0.11	362.37 ± 14.44
*t*	0.97	1.09	1.36	2.62	2.88	3.11
*P*	0.351	0.295	0.197	0.023	0.019	0.010

Note: Group A underwent retrograde tibial nailing, Group B underwent distal tibial plating.

### Torsion test

**FIGURE 5:**
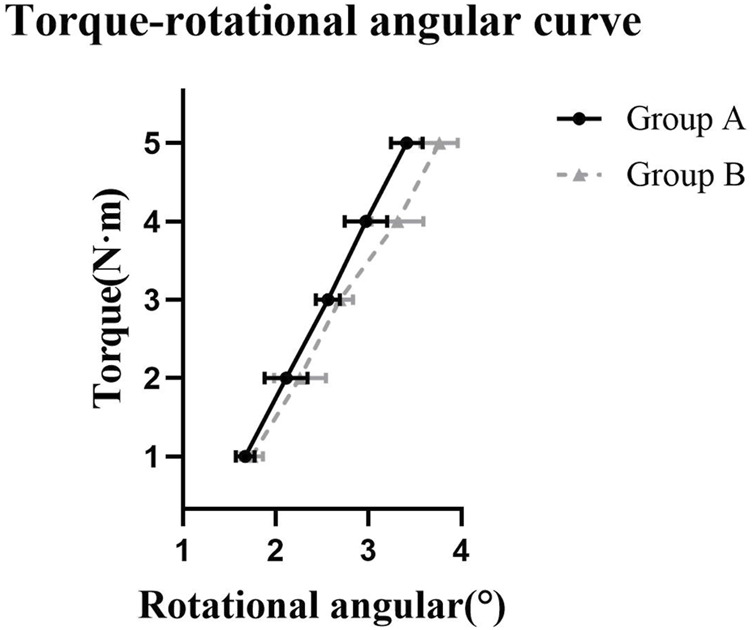
The torque-rotational angular curve for torsion test. Note: Group A underwent retrograde tibial nailing, Group B underwent distal tibial plating.

There were no statistically significant differences in torsion angles between the two groups at torque levels of 1 N m, 2 N m, and 3 N m (*p* > 0.05). However, at torque levels of 4 N m and 5 N m, Group A exhibited significantly lower torsion angles compared to Group B (*p* < 0.05). Furthermore, at a torque level of 5 N m, Group A demonstrated higher torsional stiffness than Group B, and the difference was statistically significant (*p* < 0.05). Detailed results can be found in Table 2; Figure 5.

**TABLE 2:** Torsion testing of the two groups at different torque levels.

	Torsion angles (°)					Torsional stiffness (N·m/°)
	1 N m	2 N m	3 N m	4 N m	5 N m	
Group A	1.67 ± 0.10	2.11 ± 0.23	2.56 ± 0.13	2.97 ± 0.23	3.41 ± 0.17	1.48 ± 0.07
Group B	1.74 ± 0.12	2.26 ± 0.28	2.69 ± 0.14	3.31 ± 0.28	3.76 ± 0.20	1.36 ± 0.06
*t*	1.20	1.06	1.67	2.39	3.56	3.67
*P*	0.256	0.314	0.116	0.035	0.004	0.003

Note: Group A underwent retrograde tibial nailing, Group B underwent distal tibial plating.

## Discussion

The treatment of distal metaphyseal tibia fractures continues to pose challenges and remains a topic of controversy (Iqbal and Pidikiti, 2013) Treatment for distal tibial fractures can be classified into conservative management and surgical intervention. Conservative treatment, such as plaster cast immobilization, is commonly recommended for stable fractures with no significant displacement. Surgical treatment is often preferred for unstable fractures (Newman et al., 2011). Surgical fixation options for distal tibial fractures include distal tibial plate, MIPO or intramedullary nailing. Each fixation method has its advantages and disadvantages. The key to successful surgery lies in the management of soft tissues and the preservation of blood vessels (Dickson et al., 1994). Preoperative soft tissue condition, surgical technique, and the choice of internal fixation can all influence fracture healing and increase the risk of osteonecrosis and infection (Singer et al., 1998). An optimal treatment approach should consider the pre-existing soft tissue condition and the patient’s medical history. MIPO with a medial distal tibial plate placement is often considered a viable option, although it may pose challenges in patients with compromised soft tissues, peripheral arterial disease, or diabetes. MIPO has been advocated for its potential to minimize soft tissue injury and achieve favorable clinical outcomes (Borg et al., 2004; Redfern et al., 2004; Vallier et al., 2011). However, it is worth noting that complications related to plate-induced skin irritation, skin necrosis, and plate exposure have been reported in MIPO procedures for distal tibia fractures (Lau et al., 2008; Gupta et al., 2010). In our viewpoint, the utilization of a specifically designed intramedullary implant for distal tibia fractures may offer certain advantages compared to existing options. This implant necessitates only one 2-cm-long incision for nail insertion and five 1-cm-long stab incisions for interlocking. Although placed on the medial side, which is typically associated with soft tissue injury, the modified intramedullary implant presents a less invasive alternative to MIPO plating, which requires tunneling through contused soft tissues. The intramedullary nail provides a reliable means of achieving stable fixation in the challenging distal 6 cm region, which poses difficulties in current treatment approaches. The distinctive design of the RTN enables effective management of far distal tibial fractures. By ensuring optimal placement of the nail, with the most distal locking option positioned parallel to the plafond, all available distal locking options remain within a proximity of 25 mm from the joint line.

RTN is a novel surgical option for treating distal tibia fractures that has not yet been widely adopted in clinical practice. Only a few cases have been reported in the literature (Frankle et al., 1999; Gehr et al., 2004). Utilizing a closed surgical approach with RTN appears to be a viable choice, as it allows for effective fixation of distal tibia fractures while minimizing soft tissue damage. The treatment of distal tibia fractures using RTN has undergone multiple exploratory studies in the past. The concept of intramedullary nailing for distal tibia fractures was initially introduced by Kuhn et al. (Kuhn et al., 2014c) who subsequently developed a specialized RTN for treating distal tibia fractures (Kuhn et al., 2014b). This internal fixation device is inserted into the distal end of the tibia and can be locked with multidirectional screws, providing stability for the majority of distal tibia fractures while preserving local soft tissues and blood vessels. In theory, it offers higher biomechanical stability in distal tibia fractures (Kuhn et al., 2014b).

The neutral position compression test is commonly used to assess the compressive performance of internal fixation devices. This study demonstrates that at pressures of 400 N and 500 N, the axial compression performance of RTN is superior to that of distal tibia plates. In a biomechanical comparison study conducted by Kuhn et al. (Kuhn et al., 2014b) RTN exhibited higher stability in axial compression testing when compared to an expert intramedullary nail. Another study reported similar findings in a biomechanical comparison test between RTN and minimally invasive percutaneous plates. The results showed that RTN had better stability in axial load testing, with significantly higher maximum compressive load compared to plates, and these differences were statistically significant (Kuhn et al., 2014a) The author’s analysis suggests that due to the relative intramedullary fixation of RTN compared to plates, it can reduce stress on both sides of the fracture ends, decrease shear forces, and thereby provide greater stability to withstand higher compressive forces.

The torsion test, which measures the torsional resistance of internal fixation devices, is a commonly used biomechanical performance test for lower limb fracture fixation. Greenfield et al. conducted torsional biomechanical testing on RTN and found that both triple screw locking and double screw locking demonstrated good torsional resistance capabilities (Greenfield et al., 2022) In a biomechanical comparison study conducted by Kuhn et al. between RTN and minimally invasive percutaneous plates, the results showed that RTN exhibited better stability in both clockwise and counterclockwise torsion (Kuhn et al., 2014a). The findings of this study are consistent with the aforementioned research, where RTN demonstrated superior torsional resistance performance compared to distal tibia plates at torque levels of 4 N m and 5 N m. According to the author’s analysis, the RTN used in this study employed triple screw locking at the distal end and double screw locking at the proximal end, which contributed to its ability to resist torsional forces effectively, thus providing greater stability to the fracture site.

There are still some limitations in this study. Firstly, the sample size was determined based on previous research as no pilot study was conducted (Kuhn et al., 2014b). Additionally, the study had a limited sample size, and obtaining specimens was challenging due to various limitations in conditions. Furthermore, multiple tests were conducted on the same specimen, which may introduce bias in the analysis of the measured data. Secondly, there are inevitably individual differences among specimens, leading to certain systematic errors. As RTN is a relatively new technology, there is currently limited research available, and there is a lack of large-sample randomized controlled studies. Furthermore, as these specimens were to be used for future research after this study, in order to avoid irreversible damage to the specimens, lower pressures and torques were selected (Weng et al., 2020). In addition, the frequency and duration were determined based on our previous research experience and laboratory and conditions. Regrettably, due to limitations in site and instrument conditions, no additional photographs were captured after the tests. Further in-depth research is needed to provide more comprehensive data support for its clinical application.

The results of the biomechanical testing in this study demonstrate that RTN exhibits superior performance in terms of axial compression and torsional resistance for the fixation of distal tibia fractures compared to distal tibia plates. The fixation of distal tibia fractures with RTN provides increased stability and holds promise for clinical application, leading to improved healing rates and reduced complications associated with distal tibia fractures. In summary, this study elucidates that RTN possesses significantly better biomechanical properties than distal tibia plates, providing more comprehensive biomechanical data support for its future clinical application.

## Data Availability

The raw data supporting the conclusion of this article will be made available by the authors, without undue reservation.
